# Prognosis in Pulmonary Tuberculosis

**Published:** 1906-10

**Authors:** Mary Lapham

**Affiliations:** Highlands, N. Y.


### PROGNOSIS IN PULMONARY TUBERCULOSIS.*

BY MARY LAPHAM, M.D.,
HIGHLANDS, N.C.

In the treatment of pulmonary tuberculosis it is not always possible to determine from the condition of the lungs whether the patient is gaining or losing ground. In some cases considerable time must elapse before there is sufficient change in the lung tissues for us to detect it by physical signs. And this is equally true, whether the foundations for future recovery are being laid slowly but surely, or an insidious extension of the process is burrowing and undermining and removing every hope of recovery. The area involved may be so extensive that we know it will be months before we can say just what is happening within its obscurity. We can detect a further advance, but to clear up a large area requires time, and from physical signs alone we can not always say what is happening, because the changes occur so slowly and imperceptibly. Other cases have a great deal of superficial dullness, due to pleurisy. This greatly obscures the underlying conditions, and the breath sounds and percussion notes do

*Read before the Fulton County (Georgia) Medical Society, September 20, 1906.

not always indicate the true state of affairs, which only becomes apparent after the pleurisy and superficial dullness disappear.

Then in fibroid phthisis all changes are made very slowly, and for some time it may be doubtful whether arrest or extension will prevail.

In these uncertain cases time is the greatest factor in prognosis, but while we are waiting for time to decide, and doing our utmost meanwhile to accomplish a favorable result, we are very glad to employ several prognostic methods to aid us in keeping track of the case, and to gain some opinion as to whether the patient is really gaining or losing ground.

The prognostic methods most commonly employed relate to the cough, the expectoration, the temperature, the pulse rate, the percentage of hemoglobin, the blood pressure, the quantity of phosphoric acid excreted in twenty-four hours, the opsonic index, and the differential count of the neutrophil leucocytes.

If the cough is less annoying and less frequent, and if the sputum comes away more easily and promptly and diminishes in quantity and becomes thinner, we are encouraged and inclined to hope, if not to believe, that the patient is better, even if other conditions have apparently not improved in proportion. Generally speaking, one is a good deal encouraged when the patient says the cough is better, and if at the same time the expectoration is lessened, it is very natural that we should regard this as a favorable sign. It not infrequently happens, however, that the patient says: "Doctor, I feel better, why is it that I am so much weaker? My cough is less troublesome at night, so that I sleep better, and I cough less through the day. I should think I was so much better if it were not for this weakness." Per contra, in cases where there is a good deal of pleurisy, the patient has every now and then distressing exacerbations of coughing, so violent as to cause vomiting and a condition almost paralleling whooping-cough, and the patient is greatly depressed and discouraged and says there is no hope of getting well, since the coughing is becoming so intolerable, and he may just as well go home and die there, etc. In the first case the cough is better because the patient is so much worse. Everything is stopping, and with all else the coughing and expectoration. There is, perhaps, no fever and no apparent change for the worse in the lungs, but the pulse is small and frequent, and the production of energy is so reduced that it is only a question of time when vital manifestations will be impossible. This is especially characteristic of atrophy of the gastro-intestinal tract. The patients do not die because so much more of the lung has become involved, but because not enough energy can be created to sustain life. On the other hand, in the pleuritic cases it is quite possible for the patient to improve, while the cough becomes worse. When there is a good deal of pleurisy in the paravertebral region the cough is often violently paroxysmal, and resembles that due to pleural exudates. It might almost be called tubercular whooping-cough. It occurs in paroxysms, vomiting is intimately associated with it, there is the same difficulty in removing the sputum; it is markedly worse at night, and the slightest excess in eating or drinking often results in the loss of whatever has been taken. The condition is tormenting in the extreme, and yet the patient gains steadily, the lungs may even clear up a little, while, apparently, the patient is losing ground and is most confident that he is not only much worse, but will never recover. The prognosis of these pleuritic-paroxysmal coughs is by no means bad. They may last a week or ten days, or much longer, and the patient improve steadily through it all. It is hard to say just what the prognostic value of the temperature is. We are certainly glad when the fever disappears, and the patient says: "My evening temperature has been normal for weeks." This is undoubtedly encouraging, but it must not be forgotten that cases running a temperature of 101 to 102 may improve before the gain is demonstrated by the thermometer, and, on the other hand, cases running no temperature may steadily lose ground.

It is quite customary to say that all cases showing a temperature above 100 should be kept in bed, while those at 99 may be permitted, or even encouraged, to take exercise, or at least to be dressed and sit up out of doors. It may be questioned whether temperature should be given such a controlling influence in deciding whether or not the patient shall be up and dressed. It has formerly played the leading part in determining treatment, but it is doubtful if it can much longer maintain this position. The influence of stasis in the lungs, of passive hyperemia of the affected area, is so important that we must consult other factors before allowing our patients to lightly leave the recumbent and assume the erect position simply because the temperature has become normal. The influence of fatigue is one of these factors. When any effort on the part of the patient results in a subnormal temperature, this is proof positive that such efforts must be prohibited. If the patient is lying in bed, and as a consequence of talking, or the excitement of seeing too many or too new friends, the temperature falls below normal, it will be well to accustom the patient gradually to such demands upon the strength. If after a period of inaction a short drive is taken, or walk, and the temperature falls below normal shortly after the exertion is made, care must be taken to bring the strength into proportion with the demands to be made upon it, either by waiting until the patient is stronger, or else making the walk or drive shorter. Subnormal temperatures as the result of fatigue, proscribe fatigue. I had a case of caseous pneumonia that had to be moved. The temperature had previously been about 103, and shortly after the moving the nurse was greatly pleased to report a temperature of 98, not realizing that this was fatigue, and not recovery. These subnormal temperatures, occurring as the result of fatigue, promptly follow the effort made, and unless the temperature is taken at the time, they are not detected.

The pulse rate is more affected than the temperature by the toxins of tuberculosis. It increases in frequency before there is fever, and persists long after the temperature has become normal. It is, therefore, a better guide to treatment than the temperature.

A pulse rate of over 100 says that the heart is forced to too much exertion, that the circulation should be equalized by assuming the recumbent position; that the production of heat by muscle changes should be reduced to a minimum by keeping the patient in bed, and that every tax upon the patient's energy should be avoided as much as is possible, in order that all his force may be concentrated to fight the invasion. It not infrequently happens that the pulse rate is the most, and perhaps the only, unfavorable factor. The lesions are healing. The appetite is good. There is no temperature. The patient is apparently doing well. Why not get up and stir around a little? At least sit up out in the open air. It is so depressing and weakening, this lying flat in bed. The patient is allowed to assume the erect position, cautiously at first, and not more than an hour at a time. If all goes well, and the pulse rate does not increase, the hour may be extended to two, until it is proven that the erect position is not disadvantageous.

It not infrequently happens that patients apparently profit by the change from the recumbent to the erect position. There is a certain stimulus from the apparent gain that helps a good deal towards improving the appetite and general sense of well-being. Then, after more or less time of false rejoicing, the pulse rate begins to climb up from 80 to 90 or a 100. We disregard it for a few days, hoping it will be only temporary. There is no fever, no unfavorable subjective symptoms whatever. The patient says he feels better and stronger. We allow him to remain in this fool's paradise until we discover that the phosphates in the urine are markedly increasing, that the blood pressure is falling, and the percentage of hemoglobin a little less. If we persist in disregarding these danger signals, we need not be greatly surprised if some day we find that there is a slight rise in temperature, and the patient says: "Doctor, I am afraid I have caught cold again." A little later and rales appear again, and so back to bed the patient goes until he recovers from this relapse. If we had at once been warned by the advance in the pulse rate and promptly enforced the recumbent position until the strain of the erect position could have been safely endured, we should have saved our patient the loss of many days, for all the time between the first advance in the pulse rate and the slight rise of temperature might have been spent in repelling the new invasion, instead of favoring it. By the time the temperature appears it is already very late, and what shall we say of those cases characterized by absence of fever, by lack of reaction? In these cases the pulse rate is still more important, it becomes still more imperative to keep the patient absolutely quiet in the open air until the pulse rate falls below 100, even if takes months to do so. It sometimes requires a good deal of confidence in our judgment and considerable moral courage to put a patient back to bed again, and keep him there, just because there is a slight advance in the pulse rate, and especially when there are no other unfavorable symptoms. We dread the effect upon the patient and the patient's relatives, and yet it is undoubtedly the only thing to do.

A weekly record of the hemoglobin gives considerable information. In favorable cases the percentage is usually between 60 and 70, and if the percentage becomes higher we may be very confident that the patient is doing well. A gain of five per cent. in a few weeks is very encouraging, and if thereafter the percentage steadily increases the prognosis is very hopeful, even in the face of other unfavorable symptoms. A loss of hemoglobin is never to be regarded with indifference. It is always an indication for caution, and even if the patient is enjoying short drives and spending most of the day in the erect position, it may be wise to enforce the recumbent position for a few days. Under these circumstances one is often tempted to wait a little and see whether the loss of hemoglobin may not be a matter of no importance. We may be inclined to wait for further confirmation before subjecting our patient to what must appear as an excess of caution, but if the pulse rate begins to increase in frequency we shall not be far wrong if we insist upon rest in bed at least until the pulse rate subsides. If we disregard these warnings it is quite likely that some day our patient will say: "Doctor, I do not feel quite as well to-day, I am afraid I have been overdoing; perhaps I walked or drove a little too far yesterday." If a set-back really occurs, it is usually ascribed to some indiscretion on the part of the patient, and no one knows that it was in our power to detect the beginning and prevent the culmination of processes manifesting themselves later on as a relapse.

The blood pressure in tuberculosis is apt to be low. In favorable cases it may be normal or as high as 110, by the Riva Rocci apparatus, and in unfavorable cases as low as 75. I have never seen an adult case with a blood pressure as low as 90 that did well, anything below that is of evil omen. A weekly record of the blood pressure showing a steady rise as the weeks go by is most encouraging. Inability to induce this rise is to be regretted, and a fall is discouraging. If we succeed in raising the blood pressure and then, to our surprise, the weekly examination shows a fall, even if it is a slight one, we should not disregard the warning, especially when associated with a loss of hemoglobin and increase in the loss of phosphoric acid. No matter how obscure and impossible to detect the changes may be that are causing these danger signals, it will be well to heed them and to take in sail until they improve. I had one case in which these three indications were the only signs of coming trouble. It was an ambulant case, doing extremely well. When the weekly examinations were made I found to my surprise that the blood pressure and percentage of hemoglobin had fallen, and the loss of phosphoric acid increased. I could not believe that anything serious was impending. There was no increase in the pulse rate, the temperature was normal. So I waited to see what the next week's examinations would say, and in the meantime made no change in the patient's habits. The next examinations showed a further loss, and in about four days the pulse rate began to go up. Then the patient had a set-back for three weeks, and hereafter when the weekly examinations show such a combination as loss of pressure, hemoglobin and phosphoric acid, I shall put the patient to bed while I am waiting to see what is going to happen.

In tuberculosis the loss of phosphoric acid is usually excessive, often from 10 to 15 grammes a day. The gradual reduction of this loss is one of the most gratifying points in the treatment of the disease. It shows less tissue waste, and marks the change that takes place in the proportion between loss and repair. In tuberculosis reconstruction must prevail over destruction before any gain can be made, and the reduction of the loss of phosphoric acid is one of the first indications that constructive metabolism is prevailing over destructive.

It sometimes happens that after a steady fall from fifteen to say five grammes a day, that the quantity begins to increase. If a quantitative estimate is made every few days and the amount is found to increase, this is an indication for extreme watchfulness. It may be that this loss precedes all other signs, but if the loss persists we may be certain that it will not be so very long before other signs appear. In this case, as with all other unfavorable symptoms, it is well to enforce the recumbent position until we see what is going to happen.

The prognosis of any case of tuberculosis depends almost entirely upon the power of the individual attacked to manufacture certain protective substances which are capable of rendering the tubercle bacilli susceptible of being absorbed by the leucocytes.

The opsonic index is the estimate of this capacity on the part of the leucocytes to absorb the tubercle bacilli. If it is high, the case is favorable. If it can be raised by the use of tuberculin, it is favorable. If tuberculin lowers the index it is unfavorable. The use of tuberculin should always be controlled by its effect upon the opsonic index, otherwise we are in danger of doing more harm than good. Since the difficulty of making this estimate prohibits its use by the ordinary practitioner, the local or State board of health should do it for us, just as though it were a case of diphtheria or typhoid.

Arneth of Würzburg considers the results obtained by the differential counting of the neutrophile leucocytes as characteristic not only of tuberculosis, but also of the severity of the attack. According to Arneth all the neutrophiles are classified according to the number of nuclei they contain, and these classes are named 1, 2, 3, 4, 5, just as they contain so many nuclei.

In normal blood there is a standard percentage of these classes. Of the total number of neutrophiles 5 per cent. will be mononuclears, 35 per cent. binuclears, 40 per cent. trinuclears, 16 per cent. quatronuclears and ½ per cent. of highest numbers.

The number of nuclei express the stage of development and the phagocytic capacity of the neutrophiles. The more complicated the structure, the greater is its functional capacity. In the fight against the tubercle bacilli these higher forms are the active agents of destruction and, therefore, suffer far more reduction in numbers than the simpler forms. In severe cases the complex classes entirely disappear, while the percentages of the mononuclears and binuclears are correspondingly increased, together with the lymphocytes. The blood picture of tuberculosis is that of lymphocytosis, together with an increase in the lower classes of the neutrophiles and disappearance of the higher. This shifting of the percentages from right to left, from higher to lower, is strikingly characteristic. Therefore, just as these higher classes are represented, will we form our prognosis. It is a most gratifying sign of the genuineness of an apparent recovery when these higher classes are again represented and the percentages approach the normal, until finally the eosinophiles, which are usually totally absent, again reappear.

I have seen this occur in cases in which the pulmonary condition was either extensive, or so marked, or of such a marked febroid nature, that it was difficult to map out the changes. There was practically no reaction, and apparently no change, either for better or worse, until we consulted the phosphates, the hemoglobin, the blood pressure and the differential neutrophiles. Then the evidence was often conclusive as to whether the patient was really building better than we knew or slowly and imperceptibly losing ground.

By employing these various methods we are enabled to detect the onset of a new invasion before its effects are shown subjectively, or there is an increase of temperature. When the relapse is evident much good time has been lost, and the increase has been found instead of repelled.

These methods not only enable us to form a prognosis as to the probable results of our treatment, but also enable us to detect the beginning of a new invasion at a period much preceding the manifestation upon which we ordinarily depend, such as temperature, subjective and objective. Before these manifestations appear there is a preparatory period, and if we are properly warned we can put our patients into the best condition to fight, and not lose all the period of incubation and even favor its development.

---

### DIABETES MELLITUS IN CHILDREN.

H. R. Livengood reports two cases of this nature. One child was a girl two years and six months old, the other a boy four years and five months old. The first patient was put on a strict diet. On account of vomiting and tympanites, a powder of calomel, salol and sodium bicarbonate was given. When these symptoms began to subside, iron, quinine and strychnine were substituted. At first the child seemed to improve, but unfavorable symptoms soon developed, ending in coma. Death occurred seventeen days after the writer's first visit. The second child was ill enough to need medical care for seventeen days when death occurred. A third patient, whose history is given here, was a girl of eight years old. In this case the disease lasted a little over four months. Senator and Stern give the mortality in these cases at nearly 100 per cent. —*Medical Record,* April 28, 1906.

---

### SHORT UMBILICAL CORD, DIAGNOSIS OF DURING PARTURITION.

Vetrano (*Il Policlinico*) thinks that this subject has been neglected in the text-books, though it has long been recognized as a cause of dystocia. He finds that uterine pains of constant position, posterior positions of the fetus, and hemorrhage during labor, should excite the suspicion that the umbilical cord is very short. He relates two cases where these pains and hemorrhages were present, and where the umbilical cord in each case measured but 30 centimeters.

---

SHORTENING of the shoulder, as measured from the sternal end of the clavicle to the acromion process, is significant of fracture of the clavicle.—*American Journal of Surgery.*

